# Cognitive Impairment in Opium Use Disorder

**DOI:** 10.1155/2021/5548623

**Published:** 2021-07-30

**Authors:** Hossein Sanjari Moghaddam, Behrang Shadloo, Helen Shahkhah, Abbas Tafakhori, Maryam Haghshomar, Shakila Meshkat, Vajiheh Aghamollaii

**Affiliations:** ^1^School of Medicine, Tehran University of Medical Sciences, Tehran, Iran; ^2^Students' Scientific Research Center, Tehran University of Medical Sciences, Tehran, Iran; ^3^Iranian National Center for Addiction Studies (INCAS), Tehran University of Medical Sciences, Tehran, Iran; ^4^Psychiatry Department, Roozbeh Psychiatric Hospital, Tehran University of Medical Sciences, Tehran, Iran; ^5^Iranian Center of Neurological Research (ICNR), Tehran University of Medical Sciences, Tehran, Iran; ^6^Neurology Department, Roozbeh Psychiatric Hospital, Tehran University of Medical Sciences, Tehran, Iran

## Abstract

This cross-sectional study is aimed at assessing the effects of opium use disorder (OUD) on attention, working memory, and information-processing speed. Thirty outpatients with OUD and 20 healthy controls (HCs) were assessed using a neuropsychological battery consisted of Auditory Verbal Learning Test-Revised (AVLT-R), Brief Visuospatial Memory Test-Revised (BVMT-R), Digit Forward and Backward Tests (DFT and DBT), and WAIS-R Digit Symbol Substitution Test (DSST). The most affected cognitive functions in patients with OUD were detected by DBT and DSST. However, we found no significant difference between patients according to the route of administration. Within patients with OUD, DBT score was associated with opium use quantity (OUQ) (*r* = −0.385), and DBT (*r* = 0.483) and DSST (*r* = 0.542) scores were correlated with duration of use. Our findings indicated that working memory and information-processing speed are the most affected domains of cognitive functioning. DBT and DSST could be used as brief assessments in clinical settings to screen for cognitive deficits in patients with OUD.

## 1. Introduction

Opium use disorder (OUD) is a major public health concern, associated with significant health, social, and economic consequences as well as decreased quality of life in both low- and high-income societies [1–3]. The prevalence of OUD, particularly the use of synthetic opioids, is increasingly growing in several countries [1, 2]. It has also been reported that patients being treated for OUD continue to have severe social and monetary problems (De [4]). Although opioids have historically had several therapeutic indications, they could exert profound effects on the structure and functions of the human brain, such as triggering euphoria, dizziness, mood alterations, and fine motor problems [5]. Long-term use of opioids results in physical and psychological dependence [6]. Patients with OUD might also be at greater risk of using other substances such as alcohol, cannabis, and methamphetamine, leading to worse clinical outcomes and lower quality of life [7–9]. The use of polysubstance further complicates the assessment of negative consequences of opioids, per se.

It has been suggested that a considerable portion of the problems associated with OUD have their underpinnings in neuropathological alterations in the central nervous system (CNS). These neurological changes range from anatomical alterations in the white and grey matter in the frontal and temporal regions to molecular-scale disruptions in dopaminergic transmission [10]. As a result of OUD-associated neural changes, opium users have deficits in various domains of cognitive functioning such as memory, planning, inhibition, and behavioral regulation associated with the frontal and temporal regions [11, 12]. Previous studies have been limited by concomitant use of other drugs such as alcohol, amphetamines, cocaine, and marijuana. Moreover, the amount of opium use and the route of administration have almost always been ignored, while these factors might be of significance in the severity of cognitive dysfunction in patients with OUD.

Detrimental effects of chronic and pure opium usage on attention, working memory, and information-processing speed is elusive because most studies have assessed subjects with polysubstance abuse. Furthermore, there is little, if any, information on the effects of the amount of opium usage and route of administration on the extent of cognitive dysfunction in patients with OUD. In the current study, we intend to investigate the detrimental effects of OUD on attention, working memory, and information processing speed. Moreover, the potential effects of duration of opium use, “route of administration,” and “amount of opium use” are addressed in this study.

## 2. Material and Methods

### 2.1. Study Participants

This cross-sectional study was conducted on a group of pretreatment OUD subjects, which were recruited from two specialized addiction outpatient centers, from September 2017 to May 2018. The patients were regularly visited by physicians in these centers. Neuropsychological assessments were carried out in Roozbeh Hospital, Tehran University of Medical Sciences (TUMS), Tehran, Iran. The diagnosis of OUD was first made by a psychiatrist based using semistructured interviews on the same day of neuropsychological assessment. Eligible participants were patients that consumed opium for at least 3 times per week and did not receive other substances or any medication for their addiction. The minimum and maximum of duration of use for inclusion in the study were 2 and 35 years. We did not include general population of OUDs that did not meet our inclusion criteria. All patients were examined by a trained addiction psychiatrist, and those with symptoms indicative of withdrawal or intoxication were excluded. Other exclusion criteria were (1) other drugs/substances abuse, such as psychoactive drugs, marijuana, alcohol, and other substance based on the DSM-5 criteria; (2) positive results in urine screening tests for other drugs/substances, at the day of neuropsychological assessment; (3) any coexisting neurological conditions (e.g., epilepsy, Parkinson's disease, and dementia); (4) any history of a major psychiatric disorder (e.g., major depressive disorder, and schizophrenia); (5) any history of intellectual disability (i.e., intelligence quotient (IQ) lower than 70); (6) history of brain trauma leading to loss of consciousness, seizure, or stroke; and (7) being above 65 years of age. Sex-matched healthy controls (HCs) were selected from caregivers and families of OUD patients that attended the neurology clinic of our hospital and had no history of drug/substance abuse and neurological or psychiatric disorders and were voluntarily enrolled in this study. HCs had no diagnosis of any disorder and were attending the clinic as accompaniment of their patient. They were also culturally and geographically matched with our case group. HCs with IQ scores lower than 70, an older age than 65 years were excluded. We did not match the pure IQ scores as there were higher than 70 because it could not affect the cognitive assessment. All subjects were assessed with the Wechsler Adult Intelligence Scale (WAIS-III); the subjects and the control group were matched based on IQ and education. Informed written consent was obtained from all participants prior to inclusion in the study. The current study was conducted in accordance with the Declaration of Helsinki and was approved by the Ethics Committee of Tehran University of Medical Sciences, Iran (code: IR.TUMS.REC.1396.4651).

### 2.2. Quantification of Opium Use

In order to better evaluate the role of opium use on cognitive functions, we developed an applicable index, named “opium use quantity” for quantifying the amount of total opium usage. Opium use quantity was calculated as the mean amount of daily opium usage (based on participant's self-report and estimated in grams) multiplied by the duration of use.

### 2.3. Neuropsychological Assessments

Neuropsychological assessment for this study consisted of a battery of tests designed to measure baseline verbal and nonverbal memory along with attention, working memory, and information-processing speed. Cognitive assessment was first done by psychologists of the centers and reestablished by skilled psychiatry resident who was not their physician and was blind to the cases.

#### 2.3.1. Rey Auditory Verbal Learning Test-Recognition Test (AVLT-R)

AVLT-R is administered as an index for verbal learning and recall. AVLT-R has been translated and validated in Persian language [13]. In this test, the examiner reads a list, containing 15 nouns (list A) to the subject at a steady rate of one word per second. Following the presentation of list A, the subject is required to recall and articulate as many words as he/she can, regardless of the order. This trial is repeated 5 times, and after each trial, the recalled words are recorded by the examiner as *T*1 to *T*5 scores. The sum of *T*1 to *T*5 scores is recorded as AVLT-R-*T*1 to *T*5 score. Following the 5 trials, the subject is presented with an interference list containing 15 new nouns (list B) and is asked to recall and articulate and many words as possible. After the recall of list B, the subject is immediately requested to recall the words from list A without the presentation of list A (AVLT-R-immediate recall score). After 30 minutes, the subject is again required to recall and articulate list A, and the score is recorded as AVLT-R-delayed recall score.

#### 2.3.2. Brief Visuospatial Memory Test-Revised (BVMT-R)

The BVMT-R assesses nonverbal memory by measuring visuospatial learning and recall. In this test, 6 geometric drawings are printed on an A4 paper and presented to the subject for 10 seconds. Following the presentation, the subject is requested to replicate the drawings on a blank paper as accurately as possible and in the location corresponding to that of the reference paper. The trial is repeated 3 times. The replicated designs are scored 2 if they are reproduced correctly and in the corresponding location, scored 1 if correctly replicated but in a wrong place, and scored 0 if or reproduced incorrectly or different design. The scores are recorded as BVMT-R *T*1, *T*2, and *T*3 scores. After 30 minutes, the subject is required to replicate the designs, and the score is recorded as BVMT-R-delayed recall score.

#### 2.3.3. Digit Forward and Backward Test (DFT and DBT)

DFT measures short-term auditory memory and attention, while DBT relies more on working memory skills. DFT consists of 6 items, each containing two arrays of random digits with similar length. The length of items increases by one digit, the first item being 3 digits long and reaching 8 digits at the sixth item. The examiner articulates the digits of each item at the rate of one digit per second, and the subject is required to recall the digits with the correct order. The test is discontinued, when the subject misses two successive items of the same length. The score of DFT is recorded as the maximum length of item successfully recalled before missing two successive items of the same length.

The procedure and scoring of DBT are similar to DFT. However, the subject is asked to present the reverse sequence of digits. The first item is 2-digits long, and the maximum length (sixth item) is 7 digits. Prior to the administration of DFT, the subject is appropriately instructed and completes a practice round.

#### 2.3.4. WAIS-R Digit Symbol Substitution Test (DSST)

The DSST from Wechsler Adult Intelligence Scale-Revised (WAIS-R) is a 90-second test consisting of 4 rows, each containing 25 blank squares paired with a random number from 1 to 9. Briefly, a key reference row is printed at the top of the paper, which is visible throughout the test. The reference row shows the pairing of numbers with specific symbols. The subject is instructed to fill as many symbols as possible and as fast as he/she can, corresponding to the printed numbers in the rows. Prior to the test, a practice round is completed by the subject filling 7 blank squares. In the practice round, the examiner corrects if the subject delivered an incorrect response. The final score is calculated as the number of correct responses within 90 seconds, with the maximum score being 93.

### 2.4. Statistical Analysis

Statistical Package for the Social Sciences (SPSS) version 23 was used to perform the statistical analysis. The distribution of the study variables was assessed using the Shapiro–Wilk test. We first compared demographic features and cognitive functions between OUD subjects and HCs. For this purpose, Student *T* and Mann–Whitney *U* tests were applied for the comparison of normally (age, AVLT-R-T1, AVLT-R-T1 to T5, and AVLT-R Learning) and nonnormally (the rest of neuropsychological scores) distributed continuous variables between the OUD subjects and HCs, respectively. Categorical variables were compared by Chi-square test (gender) or Fisher-exact test (education), when appropriate. Cohen's *d* was reported as the effect size of cognitive function differences, which were significantly different between groups. In the next step, one-way multivariate analysis of covariance (MANCOVA) controlling for age was implemented to control for intervariable correlations and find the cognitive variables with the largest weight and importance for discrimination between patients with OUD and HCs, which was determined by the One-way MANCOVA partial Eta squared and standardized discriminant function coefficient. Similarly, we further compared the cognitive functions between OUD subjects via oral or oral + inhalation routes. Afterward, partial correlation, controlling of confounding variables including age and education, was conducted to investigate the association between duration of use and opium use quantity (OUQ) with cognitive functions. The variables with significant differences between the subjects and HCs (i.e., DFT, DBT, and DSST) were then opted for further analysis. For this purpose, hierarchical multivariate regression was used with neuropsychological scores as the dependent variables in the OUD subject group and Standardized *β*-coefficients (95% CI), *t*, *R*^2^, and *R*^2^ change were reported. *P* value < 0.05 was considered to be statistically significant.

## 3. Results

### 3.1. Demographic and Clinical Characteristics

Thirty patients with OUD and 20 HCs participated in this study. Demographic and clinical characteristics of patients with OUD and HCs are shown in Table 1. The two groups were matched for sex and education (*P* value = 0.470 and 0.506, respectively). Patients with OUD were significantly older than HCs (*P* value = 0.004). Only 43.3% of the participants had used opium solely through ingestion, whereas the rest had both inhaled and ingested opium. The duration of use in the patients ranged from 2 to 32 years. The opium use quantity in the patients was 13.08 [7.99-22.62] (median [IQR]) gram∗year.

### 3.2. Neuropsychological Scores

**Figure 1:**
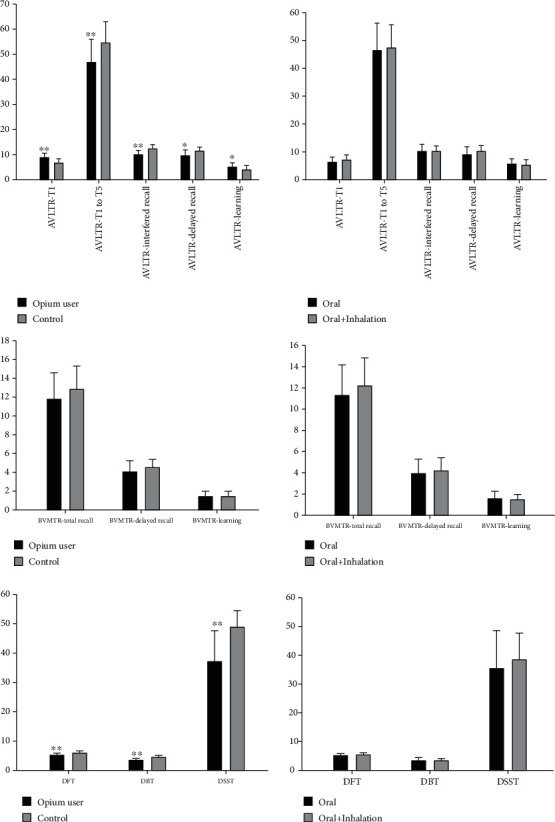
Neuropsychological scores among different study groups. AVLT-R: Auditory Verbal Learning Test-Revised; BVMT-R: Brief Visuospatial Memory Test-Revised; DFT: Digit Forward Test; DBT: Digit Backward Test; DSST: Digit Symbol Substitution Test; ^∗^*P* < 0.05, ^∗∗^*P* < 0.01.

The mean scores of patients with OUD and HCs on AVLT-R, BVMT-R, DFT and DBT, and DSST scores, are demonstrated in Table 2 and Figure 1. The results showed that patients with OUD scored lower in verbal memory tests including AVLT-R-T1 (*P* value < 0.001), AVLT-R-immediate recall (*P* value < 0.001), and AVLT-R-delayed recall (*P* value = 0.011), but no significant difference was found regarding the AVLT-R-*T*1 to *T*5 score. Moreover, the study groups showed comparable performances on BVMT-R subtest scores (*P* value = 0.212 for BVMT-R *T*1, 0.260 for BVMT-R *T*2, 0.228 for BVMT-R *T*3, and 0.240 for BVMT-R-delayed recall). Compared to HCs, patients with OUD showed significantly lower scores in DFT (*P* value = 0.007), DBT (*P* value < 0.0001), and DSST (*P* value < 0.0001).

The results of one-way MANCOVA (controlling for age) revealed that 21.2% of the difference in AVLT-R-T1, 17.6% of the difference in DBT, and 18% of the difference in DSST were due to the state of opium use (opium user or HC) (Partial Eta square = 0.212, 0.176, and 0.180, respectively). In order to control for intervariable correlations and assess the weight and importance of the tests in differentiating addicted patients from HCs, we have reported the standardized discriminant function coefficients for AVLT-R-T1, DFT, DBT, and DSST, which showed the most prominent differences between study groups. It was found that AVLT-R-T1, DBT, and DSST are the driving factors for the difference between study groups (standardized discriminant function coefficients = 0.566, 0.450, and 0.444, respectively).

Oral ingestion and smoking were the two routes of opium administration among our patients. All patients reported oral ingestion of opium, while a fraction of them reported using inhaling opium. We categorized patients into two subgroups: oral/inhalation and oral only.  Table 3 and Figure 1 illustrate the mean scores of the neuropsychological assessment between patients with OUD based on the route of administration. No significant difference was detected in any of the AVLT-R, BVMT-R, DFT and DBT, and DSST scores between these two subgroups.

### 3.3. Correlation Analysis

**Figure 2:**
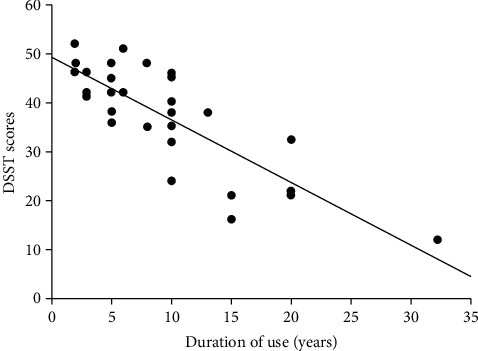
Scatter plot of the correlation between duration of use and DSST scores in opium users. DSST: Digit Symbol Substitution Test.

At the first step, we investigated the correlation between neuropsychological scores and demographic and clinical characteristics including age, education, duration of opium use, and opium use quantity in patients with OUD (Table 4). After controlling for age and education, a significant correlation was found between DBT score and the duration of opium use (*r* = 0.483 and *P* value < 0.01) and opium use quantity (*r* = −0.385 and *P* value < 0.05). Moreover, DSST score was associated with the duration of opium use (*r* = 0.542 and *P* value < 0.01) (Figure 2).

Table 4. Partial correlation (controlling for age and education) between clinical characteristics and neuropsychological scores in opium users.

### 3.4. Hierarchical Multivariate Regression Analysis

The neuropsychological tests measuring attention (DFT), working memory (DBT), and information-processing speed (DSST) were selected for further regression analysis (Table 5). Using hierarchical multivariate regression analysis, age, education, duration of opium use, and opium use quantity were included into blocks in a stepwise manner as the independent variables, and the neuropsychological scores were included as the dependent variables. Duration of opium use could significantly predict the scores of DSST (Standardized *β* − coefficients = −0.510, *R*^2^ = 0.738, *P* value = 0.003) and DBT (Standardized *β* − coefficients = −0.527, *R*^2^ = 0.618, *P* value = 0.009), explaining 10.9% of the variance in DSST score and 11.6% of the variance in DBT score.

## 4. Discussion

To the best of our knowledge, this study is among the first investigations into possible effects of chronic pure opium use on cognitive functioning of patients with OUD, controlling for the duration, average amount, and route of opium administration. Cognitive functioning was assessed using a battery of neuropsychological tests assessing verbal and nonverbal memory, attention, working memory, and speed processing. During the past decades, a number of neuropsychological studies have investigated the cognitive impairments in patients with OUD using different batteries of tests. However, the results of previous studies have been limited or confounded by several factors, which have been obviated in the current study: (1) few studies evaluated opium users, per se; as the majority of opium users consume other substances, as well. In contrast, the participants of our study were opium users only with no other concomitant substance abuse; (2) in contrast to the previous studies, which have applied a restricted battery of neuropsychological tests, we have used a constellation of tests assessing various aspects of cognition such as verbal and nonverbal memory, attention, working memory, and speed-processing; (3) the duration, amount, and route of opium use in patients, which were missing in the previous studies and might have affected the level of cognitive impairments in patients with OUD. For this purpose, these data have been included in the current study, and their possible effects have been quantified.

Several studies have reported the detrimental effects of chronic opioid use on various domains of brain functioning. Bruhn and Maage [14] compared two groups using marijuana/amphetamine/hallucinogen versus marijuana/amphetamine/hallucinogen plus opioid. They found no significant difference between two the groups based on their neuropsychological assessments. However, no information was presented about the amount and duration of opioid use, which might have had significant effects on the extent of cognitive deficits. A number of studies comparing the cognitive abilities in opioid users to those of healthy subjects reported mild impairment in different domains such as attention, visual memory recall, and visuospatial and visuomotor functions [15–19]. It has also been reported that opioid detoxification leads to improved attention, memory, and verbal fluency [20]. Lee and Pau [21] demonstrated that former heroin users behave recklessly and were ignorant of the rules and did not have good problem-solving skills. In fact, several studies have shown that heroin users have severe deficits in various executive functions, including intradimensional set-shifting, perseveration, risk-taking, and decision-making [22–25]. Subsequent neuroanatomical and neurofunctional studies suggested that grey matter atrophy in the medial and inferior prefrontal cortex, insula, and temporal cortex, along with the abnormal activation of the rostral anterior cingulate cortex are the possible underpinnings of these executive dysfunctions in heroin users [26, 27]. In contrary, there are few studies, which have reported no significant cognitive impairments among opioid users. Ersche et al. [28] stated that current and ex-users of opioids did not perform differently on neuropsychological assessments such as spatial planning, paired associative learning, and visual pattern recognition. However, about half of the ex-users had also used stimulants, which may be the reason why no difference was found between current and past opioid users. Furthermore, a recent prospective study investigated the effects of prescription opioids on cognition but reported no detrimental effect [29].

Overall, previous studies on opioid users point toward impairments in different domains of cognition; nevertheless, their results are confounded by the use of drugs other than opioids. Few studies have investigated the effect of duration of use, level/amount of opioid use, and route of opioid administration (oral, inhalation, or injection) on the cognitive functions in patients with OUD. Addressing these liabilities, we found that patients with pure OUD have deficits in specific aspects of brain functions, represented by AVLT-R, DBT, and DSST. In fact, our results showed that AVLT-R-T1, DBT, and DSST are the deriving evaluations for differentiating opium users and HCs. However, the effect size for these differences was quite small. We, further, intended to investigate the role of two important factors: “route of administration” and “the amount of opium use” on the severity of cognitive impairments in patients with OUD. Our findings revealed no difference between patients with OUD in “oral/inhalation” or “oral only” groups. However, OUQ was associated with lower scores in DBT among patients with OUD. Moreover, we found that the duration of use is significantly correlated with the scores of DBT and DSST. In this regard, DBT and DSST could be used as brief and useful assessments in clinical settings to screen for deficits in working memory and speed-processing in patients with OUD. Nonetheless, it should be noted that deficits in working memory and speed-processing are not specific to a single disorder, since they can occur in different neurological and psychiatric disorders. Moreover, no other “frontal-executive” neuropsychological tasks assessing intra/extradimensional set shifting, planning and decision making, and logical-perceptual reasoning have been administered in this study.

Digit span tests (DFT and DBT) are applicable and useful neuropsychological assessments in clinical settings, widely utilized for the evaluation of cognitive disorders such as dementia and depression [30–34]. Attention and working memory are the main domains of the brain functions assessed by DFT and DBT. DBT involves manipulating the numbers and, thus, is more sensitive to cognitive impairments [31, 35]. We observed that patients with OUD had deficits in DBT, but not in DFT, which might suggest that opium use exerts more severe effects on higher-level functions of the brain. In addition, satisfactory performance on DSST requires normal processing speed, inhibition, and shifting [36], and it has been advocated that the performance on DSST is critically dependent on fronto-subcortical circuitry [37, 38]. In fact, impairment on executive processes relies not only on the prefrontal areas but rather on a complex fronto-subcortical circuitry involving thalamus, anterior cingulate, striatum, and the parietal cortex.

Our study was limited by certain factors. The small sample size of this study was the main limitation, which was due to the fact that only pure opium users were included in this study. Small sample size is generally associated with lower statistical power of study to detect between-group differences. Therefore, in this study, we did not correct for multiple comparisons, which would further affect the power of our study. Although we found no significant effect for the “route of administration” on neuropsychological scores, this might be due to the small power of the study. Thus, studies with larger sample sizes should address this issue. Second, the inclusion of only pure opium users might restrict the generalizability of our findings regarding addictions to other opioid substances and prescription analgesics. However, this can be due to the fact that in Iran, most of the patients, especially older adults, consume opium only. This may have cultural reasons; for example, some of them consider opium as an advantageous substance specially in some specific regions, and this type of addiction is not as faulty as other substances. One of our implications for this study was this false belief that opium consumption has favorable effects on body and cognition in the elderly. Patients usually search for treatment when medical, familial, or social complications of addiction come through. Proof of only opium using was confirmed by taking a history from themselves and their family, positive opium and negative amphetamine, cocaine, and cannabis in urine samples. The other limitation that restrict the generalizability of our findings is that we did not explore all cognitive domains. Although we included a battery of well-known and reliable tests to assess memory, attention, speed processing, and executive functions, the battery did not encompass other cognitive domains, like verbal fluency, logical reasoning, similarities, visuo-constructional praxis, and screening tests for global cognition. Fourth, this is a baseline cross-sectional study, and we could not follow our patients after treatment. Future studies should focus on the effects of different treatment modalities on the impairments of OUD patients in memory, working memory, and information-processing speed. Lastly, we used self-report to calculate OUQ and determining the route of administration.

## 5. Conclusions

In summary, we have investigated the severity of cognitive impairments in a group of patients with OUD using a neuropsychological battery consisting of AVLT-R, BVMT-R, DFT and DBT, and DSST. Our study is the first of its kind evaluating the effects of “route of administration” and “opium use quantity” on various domains of brain functions among opium users. We demonstrated that patients with OUD have impairments in verbal memory, working memory, and information-processing speed. Route of administration and OUQ had no significant effect on the severity of cognitive impairment. However, the duration of use showed significant associations with the scores of DBT and DSST. DBT and DSST could be used as office-based assessments to screen impairments in working memory and information-processing speed in patients with OUD.

## Figures and Tables

**Table 1:** Demographic and clinical characteristics of opium users and HCs.

	Opium users (*n* = 30)	HCs (*n* = 20)	*P* value
Age (mean ± SD)	40.63 ± 12.01	31.10 ± 9.07	0.004^a^
Gender (male/female)	25/5	15/5	0.470^b^
Education (%)			0.506^c^
Not educated	1 (3.33%)	0 (0%)	
Elementary	6 (20%)	2 (10%)	
Middle school	1 (3.33%)	2 (10%)	
Diploma	11 (36.66%)	5 (25%)	
College student	4 (13.33%)	6 (30%)	
Bachelor student	7 (23.33%)	5 (25%)	
Duration of use (median [IQR])	9 [5-10.75]		
Route of opium usage (%)			
Inhalation/oral	17 (56.6%)		
Oral	13 (43.3%)		
Opium use quantity (median [IQR])	13.08 [7.99-22.62]		
Lead level (median [IQR])	4.4 [3-6.8]^∗^		

^a^Student *T*-test. ^b^Chi-square test. ^c^Fisher-exact test. ^∗^*n* = 27.

**Table 2:** Scores of AVLT-R, BVMT-R, DFT, DBT, and DSST in opium users and HCs.

	Patients (*n* = 30)	HCs (*n* = 20)	*P* value	Cohen's *d*	One-way MANCOVA Partial eta square	One-way MANCOVA Standardized discriminant function coefficients
AVLT-R-*T*1 (mean ± SD)	6.66 ± 1.66	8.90 ± 1.55	<0.0001^a^	1.39	0.212	0.566
AVLT-R-*T*1 to *T*5 (mean ± SD)	46.90 ± 8.92	54.70 ± 8.44	0.003^a^	0.89	0.041	
AVLT-R-interfered recall (median [IQR])	10 [8-11.25]	12 [11.25-14]	<0.001^b^	1.17	0.106	
AVLT-R-delayed recall (median [IQR])	10 [7-11.25]	12 [10-12]	0.015^b^	0.79	0.017	
AVLT-R learning (mean ± SD)	5.10 ± 1.97	3.90 ± 1.33	0.021^a^	0.71	0.147	
AVLT-R forgetting (median [IQR])	2 [1-3]	1.5 [1-2]	0.056^b^		0.011	
AVLT-R percent forgetting (median [IQR])	0.166 [0.122-0.276]	0.116 [0.072-0.166]	0.027^b^	0.55	0.010	
BVMT-R total recall (median [IQR])	12.5 [10.5-14]	14 [11.25-14]	0.169^b^		0.009	
BVMT-R-delayed recall (median [IQR])	4 [3-5]	5 [4-5]	0.240^b^		0.007	
BVMT-R learning (median [IQR])	1 [1-2]	1 [1-2]	0.963^b^		<0.001	
DFT (median [IQR])	5 [5-6]	6 [5.25-6]	0.007^b^	1.42	0.053	-0.250
DBT (median [IQR])	4 [3-4]	4.5 [4-5]	<0.0001^b^	0.89	0.176	0.450
DSST (median [IQR])	39 [32-46]	48 [45-53]	<0.0001^b^	1.37	0.180	0.444

^a^Student *t*-test. ^b^Mann–Whitney test. AVLT-R: Auditory Verbal Learning Test-Revised; BVMT-R: Brief Visuospatial Memory Test-Revised; DFT: Digit Forward Test; DBT: Digit Backward Test; DSST: Digit Symbol Substitution Test; MANCOVA: one-way multivariate analysis of covariance.

**Table 3:** Scores of AVLT-R, BVMT-R, DFT, DBT, and DSST in oral users and oral + inhalation users.

	Oral + inhalation (*n* = 18)	Oral (*n* = 12)	*P* value
AVLT-R-*T*1 (mean ± SD)	7 ± 1.64	6.16 ± 1.64	0.185^a^
AVLT-R-*T*1 to *T*5 (mean ± SD)	47.38 ± 8.13	46.16 ± 10.32	0.720^a^
AVLT-R-interfered recall (median [IQR])	10 [8.75-11]	10.5 [8-12]	0.755^b^
AVLT-R-delayed recall (median [IQR])	10 [8-12]	9.5 [6.25-11]	0.368^b^
AVLT-R learning (mean ± SD)	5 ± 2.02	5.25 ± 1.95	0.740^a^
AVLT-R forgetting (median [IQR])	2 [1-3]	2.5 [2-3]	0.368^b^
AVLT-R percent forgetting (median [IQR])	0.160 [0.081-0.272]	0.218 [0.142-0.321]	0.368^b^
BVMT-R total recall (median [IQR])	13 [11-14]	11.5 [8.25-14]	0.465^b^
BVMT-R-delayed recall (median [IQR])	4.5 [3-5]	4 [2.25-5]	0.545^b^
BVMT-R learning (median [IQR])	1 [1-2]	1 [1-2]	0.787^b^
DFT (median [IQR])	4.5 [3-5]	4 [2.25-5]	0.522^b^
DBT (median [IQR])	13 [11-14]	11.5 [8.25-14]	0.465^b^
DSST (median [IQR])	2 [1-3]	1 [1-2]	0.200^b^

^a^Student *t*-test. ^b^Mann–Whitney test. AVLT-R: Auditory Verbal Learning Test-Revised; BVMT-R: Brief Visuospatial Memory Test-Revised; DFT: Digit Forward Test; DBT: Digit Backward Test; DSST: Digit Symbol Substitution Test.

**Table 4:** Partial correlation (controlling for age and education) between clinical characteristics and neuropsychological scores in opium users.

	AVLT-R-*T*1	AVLT-R-*T*1 to *T*5	AVLT-R-interfered recall	AVLT-R-delayed recall	AVLT-R learning	AVLT-R forgetting	AVLT-R percent forgetting	BVMT-R-total recall	BVMT-R-delayed recall	BVMT-R-learning	DFT	DBT	DSST
Duration of use	-0.158	0.035	0.118	-0.110	0.261	0.258	0.256	-0.319	-0.122	-0.034	-0.240	-0.483^∗∗^	-0.542^∗∗^
Opium use quantity	-0.136	-0.055	-0.079	0.048	0.135	-0.001	0.019	0.088	0.069	0.130	-0.258	-0.385^∗^	-0.113

^∗^ *P* < 0.05; ^∗∗^*P* < 0.01. AVLT-R: Auditory Verbal Learning Test-Revised; BVMT-R: Brief Visuospatial Memory Test-Revised; DFT: Digit Forward Test; DBT: Digit Backward Test; DSST: Digit Symbol Substitution Test.

**Table 5:** Hierarchical multivariate regression with DFT, DBT, and DSST scores as the dependent variables in opium users.

	Variable	DSST				DBT				DFT			
		Standardized *β*-coefficients (95% CI)	*t*	*P* value	*R* ^2^ (*R*^2^ change)	Standardized *β*-coefficients (95% CI)	*t*	*P* value	*R* ^2^ (*R*^2^ change)	Standardized *β*-coefficients (95% CI)	*t*	*P* value	*R* ^2^ (*R*^2^ change)
Block 1	Age	-0.708 (-0.873 to -0.387)	-5.30	<0.001	0.501 (0.501)	-0.634 (-0.60 to -0.022)	-4.33	<0.001	0.401 (0.401)	-0.506 (-0.049 to -0.10)	-3.10	0.004	0.256 (0.256)
Block 2	Age	-0.333 (-0.606 to 0.014)	-1.95	0.061	0.629 (0.128)	-0.301 (-0.046 to 0.007)	-1.52	0.138	0.502 (0.101)	-0.051 (-0.028 to 0.022)	-0.24	0.809	0.443 (0.187)
	Education	0.518 (1.204 to 6.149)	3.05	0.005		0.460 (0.029 to 0.445)	2.33	0.027		0.628 (0.093 to 0.492)	3.01	0.006	
Block 3	Age	-0.248 (-0.491 to 0.049)	-1.68	0.105	0.738 (0.109)	-0.213 (-0.038 to 0.010)	-1.19	0.242	0.618 (0.116)	-0.005 (-0.025 to 0.025)	-0.024	0.981	0.475 (0.032)
	Education	0.195 (-1.181 to 3.943)	1.10	0.178		0.125 (-0.160 to 0.289)	0.59	0.560		0.452 (-0.028 to 0.449)	1.81	0.081	
	Duration of use	-0.510 (-1.305 to -0.301)	-3.28	0.003		-0.527 (-0.104 to -0.016)	-2.81	0.009		-0.277 (-0.075 to 0.018)	-1.26	0.219	
Block 4	Age	-0.266 (-0.517 to 0.043)	-1.74	0.094	0.742 (0.004)	-0.167 (-0.035 to 0.013)	-0.928	0.362	0.642 (0.024)	0.035 (-0.024 to 0.028)	0.16	0.870	0.493 (0.018)
	Education	0.200 (-1.185 to 4.022)	1.12	0.272		0.112 (-0.165 to 0.280)	0.53	0.599		0.440 (-0.034 to 0.445)	1.76	0.090	
	Duration of use	-0.548 (-1.412 to -0.312)	-3.22	0.003		-0.430 (-0.096 to -0.002)	-2.15	0.041		-0.192 (-0.070 to 0.031)	-0.81	0.426	
	Opium use quantity	0.081 (-0.136 to 0.244)	0.58	0.563		-0.211 (-0.026 to 0.006)	-1.29	0.206		-0.183 (-0.025 to 0.009)	-0.94	0.352	

DFT: Digit Forward Test; DBT: Digit Backward Test; DSST: Digit Symbol Substitution Test; MANCOVA: one-way multivariate analysis of covariance.

## Data Availability

Data is available upon request.
